# MoFlow: visualizing conformational changes in molecules as molecular flow improves understanding

**DOI:** 10.1186/1753-6561-9-S6-S5

**Published:** 2015-08-13

**Authors:** Shareef M Dabdoub, R Wolfgang Rumpf, Amber D Shindhelm, William C Ray

**Affiliations:** 1 The Ohio State University Department of Periodontology, Columbus, OH, USA; 2 The Research Institute at Nationwide Children's Hospital, Columbus, OH, USA

**Keywords:** Molecular Dynamics, Molecular Structural Biology, Visualization, Molecular Conformation

## Abstract

**Background:**

Current visualizations of molecular motion use a Timeline-analogous representation that conveys "first the molecule was shaped like this, then like this...". This scheme is orthogonal to the Pathline-like human understanding of motion "this part of the molecule moved from here to here along this path". We present MoFlow, a system for visualizing molecular motion using a Pathline-analogous representation.

**Results:**

The MoFlow system produces high-quality renderings of molecular motion as atom pathlines, as well as interactive WebGL visualizations, and 3D printable models. In a preliminary user study, MoFlow representations are shown to be superior to canonical representations for conveying molecular motion.

**Conclusions:**

Pathline-based representations of molecular motion are more easily understood than timeline representations. Pathline representations provide other advantages because they represent motion directly, rather than representing structure with inferred motion.

## Background

### Introduction

Few proteins are as conformationally rigid as their "structures", accessible through the Protein Data Bank (PDB), would imply. To be sure, PDB structure files often contain confidence-level information detailing how much uncertainty is present in the coordinates of the individual atoms, or contain several alternative structures implying that the protein can transition between them. However, canonical representations for molecular structures and their mobility convey none of this information to the user in an intuitive fashion. The atoms of proteins are represented as having single discrete locations and colored by confidence, or the molecule is represented as having a set of discrete, perfectly-known poses, with no representation of transition between them.

Fluently reading these representations requires considerably user training and experience because they are highly reliant on convention. Molecular biologists who live with them daily do develop facility, but the end-customer biologist is often left bewildered because the representations eschew sensory cues.

In this manuscript we introduce a sensory-based representation for molecular motion that is aimed at the end-customer basic bio/life-sciences researcher who wants to understand "how did my molecule move", and who wants to be able to make intuitive comparisons between different molecules, without becoming deeply experienced with quantitative molecular biology tools. We also introduce a web resource that enables users to construct these representations from their own data, in a 2D projection form, a 3D virtual model form, and also a 3D-printable form.

### Justification for the need

As more detail is uncovered about protein structures and their flexibility, it is often found that the dynamics of conformation changes - not simply the alternate conformations themselves, but the molecular motion, the paths taken between conformations and the frequency with which different molecules take different paths - play a part in modulating the molecular functionality [1]. This phenomenon is a source of fascination for the molecular biologist who wishes to understand how and why the dynamics affect function, but it is a frustration for the virologist who wants to know whether an antibody blocks a folding event, the gene therapist who wants to know where she can modify a delivery vector without altering its necessary mechanics, and the microbiologist trying to understand how a seemingly innocuous change to an anti-microbial peptide produces a surprisingly large change in efficacy. Current analytical methods are producing ever more data that can help answer questions about conformational flexibility for these non-molecular-biologist users, yet current visualization tools are targeted to the quantitative molecular biologist, rather than their end-customer who needs to understand, rather than measure the motion.

These users specifically need:

• To quickly acquire a qualitative overall understanding of a molecular motion: "It moved like this".

• To quickly identify regions of significant motion and of little motion: "This moved, that didn't".

• To differentiate (largely) ordered motion from (largely) unordered motion. "It moved from here to there", versus "it jiggled about".

• To understand timing and speed: "It spent a long time, here, then quickly moved there".

• To understand the similarities and differences between different molecular motions (of the same molecule, or between different molecules): "These all moved similarly, while that mutant takes a different path".

Addressing these needs requires a representation that promotes motion to the primary feature being conveyed, rather than representing conformations as the primary feature and requiring interpretation to recognize motion.

### Typical data and representations

Nuclear Magnetic Resonance imaging (NMR) or Molecular Dynamics simulations (MD) can yield snapshots of molecular conformations that represent changes in the structure of a molecule over time. Traditionally, visual representations of molecular motion have consisted simply of displaying these snapshots as a series of static images, often overlaid upon each other, similar to stroboscopic multiple-exposure pictures of motion [2]. In such representations, each overlaid pose represents the physical state of the molecule at a discrete moment in time.

Such representations can be used in full 3D interactive modalities, as well as in 2D modes, and can easily be animated by simply playing each pose in sequence. Unfortunately, they are not well suited for conveying molecular motion and variations in molecular motion to users. Instead of representing motion, they represent poses - motion must be inferred. If a color gradient is used to convey sequential position in the molecule (as is commonly done in single-conformation images), then conveying temporal order for the poses becomes problematic. If color is used to convey temporal order, then sequential position in the molecule is lost. Even more problematically, if the motion is dramatic, some parts of the molecule may move so far between poses that the visual ordering becomes disjoint despite coloring, and understanding the motion between sequential poses becomes difficult for the viewer despite the temporal coloring scheme.

We propose that a large part of the problem with this pose-based representation is that it is effectively orthogonal to the most intuitive representations for conveying motion. The pose-based representation is a direct equivalent to Timeline representations of fluid flow. As an alternative, effectively time-lapse representation, we propose using an analog to Pathline representations for molecular motion. While Wang *et al*. [3] identified little difference in user utility between Timelines and Pathlines for understanding motion, our experience with user-consumers of molecular motion information, and our data comparing these representations, suggest that pure timeline representations are distinctly suboptimal for representing molecular motion.

Of critical importance, this simple transformation from sequential poses of the structure to integral paths for atoms over time converts *motion *into a structure that is directly represented, rather than requiring the user to infer motion from representations of *molecular *structures. This has both immediate consequences for understanding, as well as larger consequences for possible extensions to the representational paradigm.

MoFlow, our web-based system for generating time-lapse molecular-flow images and motion structures, accepts multi-pose Protein Data Bank (PDB) files, and creates an interactive WebGL visualization of the molecular motion, a renderable POV-Ray scene file, and a color-per-vertex VRML file that is suitable for printing on a 3D printer.

In this manuscript we introduce the MoFlow system, which was developed to explain the movements of a particular molecule to a collaborator working on gene-therapy constructs, and present an initial user study comparing user-understanding for MoFlow molecular representations compared to typical pose-based representations, with a broad-based user population.

The MoFlow webserver can be accessed at: http://www.mathmed.org/moflow/

### Related work

There is a clear parallel between Molecular motion and fluid motion, and importantly, between the results of molecular motion simulations and Computational Fluid Dynamics (CFD) simulations. This parallel strongly suggests that visualization methods that have been found successful for fluid flow data, may be similarly useful for visualizing molecular motion. There are however, subtle differences between molecular motion and fluid motion, and in the questions that one might wish to answer with each.

The molecular motion with which we are interested is that of individual atoms in an individual molecule. At any given point in time, these atoms have one and only one location. These locations change over time, and so Pathlines for each atom can be traced. Likewise Timelines for the molecule correspond to the backbone pose conformation at different times. However, unlike fluid flow, there are no atoms "further along", or "following behind" the atoms of the molecule being examined. As a result, flow concepts such as Streaklines do not apply. Also, because each atom has only a single velocity, and exists at only a single point at any instant in time, Streamlines devolve to vector stream tangents, and do not imply a "bulk" motion as they do in fluid.

An additional important difference derives from the fact that the atoms of interest in the molecule, have a distinct sequential ordering and connectivity that is meaningful to the user and must be represented.

CFD visualization methods have previously been applied to some types of Molecular Dynamics simulation data. Schmidt-Ehrenberg *et al*. [4] generated isosurfaces from conformational densities of metastable molecules, showing a convex hull of the range of "fuzzy" molecular conformations. Bhattarai and Karki [5] used time-gradient pathlines to represent atomic positions in a simulated closed-system liquid solution. Similarly, Bidmon *et al*. [6] used pathlines representing whole molecules (e.g. water) colored on a gradient indicating time to visualize whole-system dynamics of solvents near protein cavities. In this study, varying levels of detail were achieved by the application of a clustering algorithm to smooth finer details of motion. Falk *et al*. [7] also use pathlines for whole proteins to understand the micro-scale fluid-like motion and transport of proteins within cells. They introduce some simplifications involving recasting particle data as volume data, and exploiting the repetitive structure for reducing the enormous complexity of whole-cell simulations. Finally, Joshi and Rehingans [8] proposed a number of illustration-style techniques for visualization of general time-varying data. These included speedlines as smoothed/low-detail versions of particles traces, flow ribbons (essentially paired speedlines) to occlude minor detail and impart an overall impression of motion, opacity modulation to indicate direction of time, and strobe silhouettes to abstractly convey previous positions of objects.

Unfortunately for the researcher wishing to understand the motion of a macromolecule, such as a protein, undergoing a conformational change, none of these approaches deal with the composite trajectory of the backbone structure for a single molecule. Instead they have dealt with related concepts such as the paths of multiple molecules in solution, or the envelope of conformations that a particular molecule may adopt.

Surprisingly, one of the most promising existing data analogs, and visualization approaches, is found in the analysis of dance, by Forsythe [9], where representation of the positions and poses of a dancer, as well as the motions of their limbs over time, are critical to visualizing dance choreography. In protein motion however, the presence of a backbone creating a representationally-critical ordering, and distinct spacing for the atoms in a protein, as well other unique features, makes protein conformational change sufficiently different as to require a distinct approach.

While animation would seem an obvious means of addressing the problem of visualizing conformational change, a simple amalgamation of static poses into an animation makes the motion easy to understand, but makes it impossible to directly compare different stages of the motion. Non-sequential time points are difficult to compare due to change-blindness and inattention-blindness effects [10]. The human brain can rarely accurately perceive and recall any objects other than the few it visually focused on, and rarely recalls original states, let alone transitional states. As such there is a clear need for a technique by which molecular motion can be represented as a static image which is able to convey the dynamic behavior of a molecular in a manner which the human brain is capable of perceiving.

## Methods

### MoFlow intention and design

MoFlow was originally developed to assist a collaborator who was trying to engineer novel cell-targeting motifs into the Adeno-Associated Virus Type-2 (AAV2) capsid protein. AAV2 is a nearly ideal candidate for a gene-therapy delivery vector, but suffers from several issues that make its direct application problematic; first, many people have already developed immunity to AAV variants, and second, AAV2 is reasonably non-specific about the cells it infects, making it difficult to use for delivering therapy constructs to targeted cell types. Removing epitopes that most peoples' immune systems recognize from the capsid (the viral protein coat) surface, and adding targeting motifs that recognize specific cell-surface receptors would be relatively straightforward, except for a confounding feature of the AAV2 capsid: it is both a packaging unit, and a machine with an enzymatic functionality required in the lifecycle of the virus.

Research suggests that conformational changes of the capsid are a required feature of its lifecycle - specifically it appears that a conformational change induced by acidification of the endosome causes a phospholipase catalytic domain to be exposed, enabling the virus to escape the endosome. Therefore changes to the capsid, such as altering existing epitopes and adding new targeting factors, must be done without significantly affecting the ability of the capsid to undergo the conformational changes required to expose the phospholipase domain.

Molecular dynamics simulations were conducted to identify how the AAV2 capsid protein moves during acidification, but while the results of these simulations were reasonably easy to understand for expert users of MD software, our gene-therapist collaborator who needed the information found all attempts to convey the results using typical molecular visualization software such as PyMol [11] and VMD [12] to be uninterpretable.

Recognizing that the primary difficulty was due to the "timeline" representation of overlayed poses used by typical MD visualization software, we developed MoFlow as an alternative. The understanding gained from our alternative representation resulted in several successful engineered AAV2 capsid alternatives reported in Davis et. al. [13]

### MoFlow flow visualization

MoFlow accepts a time-ordered series of molecular "poses", typically produced by a molecular dynamics simulation, or by a conformational-morph prediction such as is produced by the Yale Morph server [2].

From this ordered series of poses, MoFlow extracts the backbone alpha carbons and interpolates intermediate positions for each of these by constructing a natural cubic spline that passes through each of that carbon atom's predicted positions in order, over time. This spline is a smoothed Pathline for that atom. Different choices of temporal resolution in the input series of poses, and an interface control that enables the user to render only a subset of input poses, control the degree of smoothing.

On-screen, and in our printable output, this spline is rendered as a piecewise-continuous cylinder extruded along the spline. Use of the alpha carbon trace of the molecule captures significant conformational motion, while effectively acting as a low-pass filter to eliminate the high-frequency motions of amino acid side-chains. However, the representation can also be used for other atoms in the molecule, revealing, for example, concerted motion in side-chains, rather than conformational motion in the backbone. A time-based color gradient (blue through red to yellow, by default) is applied to each pathline.

For a single user-chosen time point in the simulation, the alpha-carbon trace for the protein backbone is also projected onto the pathlines. Spheres are positioned to represent the alpha carbons in a single pose (at a single timepoint), and cylinders are used to connect sequentially neighboring alpha carbons. This single molecular backbone is a single-point timeline for the molecule. This backbone provides structural context for the paths: for each atom, the pathline projects back into the past where the atom came from, and into the future where the atom is going.

In the POVRay version, the linking-cylinder between every other sequential pair of atoms in the single molecular structure drags a fading transparent membrane behind it, similar to the integral-surface ribbons introduced by Hultquist [14]. This membrane both aids in visually following the sequence of the pathlines, as well as providing an additional cue to the direction of time. The WebGL version does not currently display this membrane, and because few 3D printers can convey transparency, the printable version substitutes a "ladder rail" structure between pathlines that provides some pathline ordering context, and additionally supplies the structure necessary to hold the 3D-printed pathlines in appropriate relationship to each other.

**Figure 1:**
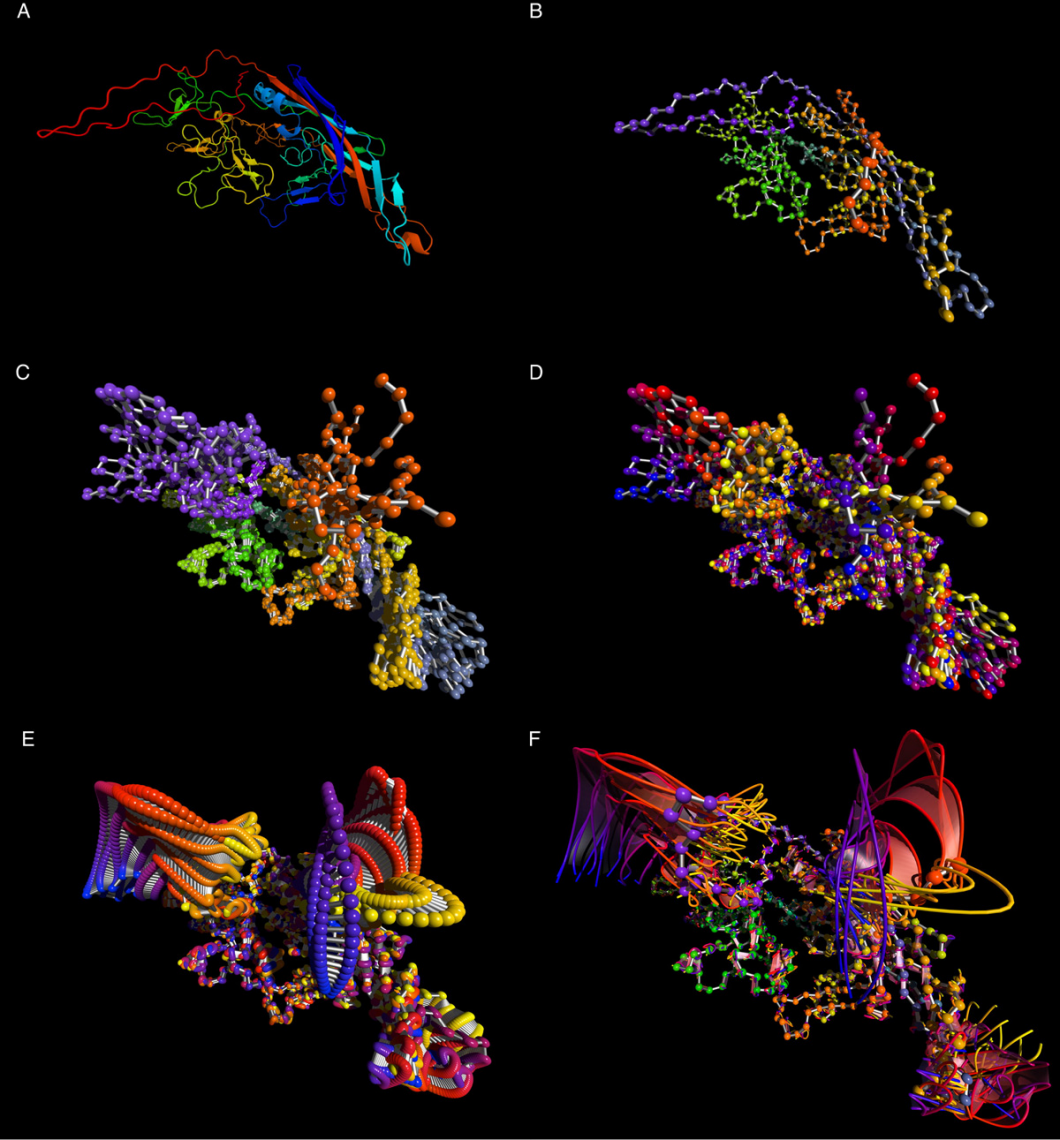
**Molecular motion becomes easier to understand as the representation becomes more flow-like**. (a) traditional cartoon representation of the AAV-2 capsid protein monomer; (b) single-pose representation of the alpha-carbon backbone of AAV-2 monomer where color represents location along backbone; (c) 10-pose rendering colored by sequential position of the molecule; (d) 10-pose rendering colored by time-point; (e) 100-pose rendering colored by time-point. (f) Full MoFlow representation. The eye is immediately drawn to the three areas of significant motion; The backbone structure remains visible, while motion paths are comprehendible with minimal effort.

Figure 1 shows a comparison between different representations of the AAV2 capsid and the motion it is predicted to perform during acidification. Figure 1a and 1b provide canonical representations of single poses of the AAV-2 capsid protein for reference, with 1a showing a typical cartoon version, and 1b showing an alpha-carbon trace rendered in MoFlow's default backbone color scheme. Figure 1c shows the alpha carbon trace of each 10 sequential poses of AAV-2, using the same sequence-based coloring scheme. This coloring still enables sections of the molecule to be distinguished, but prevents temporal ordering from being conveyed. Figure 1d shows the same sequential poses, with each pose (entirely) based on where it is located in the temporal gradient. This makes motion easier to follow but obscures structure. Finally, in Figure 1e, 100 poses are shown using the temporal, rather than sequential coloring scheme. It is easy to see that the closely spaced poses, with temporal coloring, makes the motion of the molecule far easier to discern than the more distantly spaced poses with sequential coloring. The representation in 1e has become so dense that it is impossible to see many details in it, but its success at conveying motion is a step in the right direction.

Recognizing that this success derives from the fact that the spheres representing atoms have become so dense that they are visually interpreted as being continuous pathlines, we developed MoFlow to represent pathlines for each atom in a sufficiently sparse fashion as to enable the user to "see into" the motion, and to also enable the display of molecular backbone structure onto the pathlines. Figure 1F shows an individual molecular structure, colored again as shown in 1B, with Pathlines for the atoms imposed upon it, using the coloring as shown in Figure 1E.

**Figure 2:**
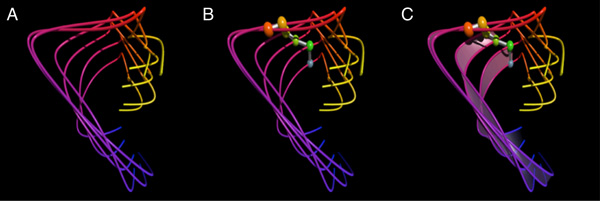
**The basic elements of a Moflow visualization representing the motion of a six-atom subset of the AAV-2 capsid protein monomer**. (a) The motion of each alpha carbon atom is represented by a natural cubic spline. The set of 3D coordinates over time for each atom define the control points. Each pathline is colored on a gradient from blue (start of trajectory), through red, to yellow (end of trajectory. (b) Added to the initial visualization is a set of spheres representing the position of the alpha carbon atoms for one pose of the molecule at a user-selectable time. (c) The full Moflow visualization for these atoms includes a set of semi-transparent ribbons between every other pair of atom pathlines. These ribbon-surfaces aid in visually ordering the pathlines as well as provide a motion cue helping the user to recognize the direction of motion.

Figure 2 provides a simple example of this representation. The six splines correspond to six atoms from an MD simulation of a small section of the AAV2 capsid monomer protein. Each of the splines is colored along a temporal gradient (in this example the gradient runs from blue through red to yellow) as an indication of increasing time in the simulation data. In part 2b of Figure 2, the next piece of the visualization has been added: a single pose of the molecular backbone represented by spheres positioned at each alpha carbon, with connecting cylinders tying together sequential alpha carbons. These alpha carbons are colored using a color gradient based on sequential ordering (here, from red through green to blue - this choice avoids overlap with the pathline colors except for at the endpoints). Inclusion of these alpha-carbon spheres provides both a specific molecular structure in the visualization, as well as providing a visual cue to the sequential ordering of the pathlines.

An additional visual cue we have added to the visualization is a set of fading, semi-transparent ribbons connecting alternate sequential atom pathlines. These ribbons "trail behind" the molecular structure backbone that is imposed on the pathlines, and provide a directional cue for the motion. By creating an oriented surface between adjacent pathlines, the direction of the motion along the pathlines becomes discernible.

Figure 2c shows how the ribbons contribute to the perception of motion beginning near the bottom and sweeping forward, then up and around to reach the provided molecular structure. At this level of detail the motion of each atom is clearly visible and easily tracked by the eye.

**Figure 3:**
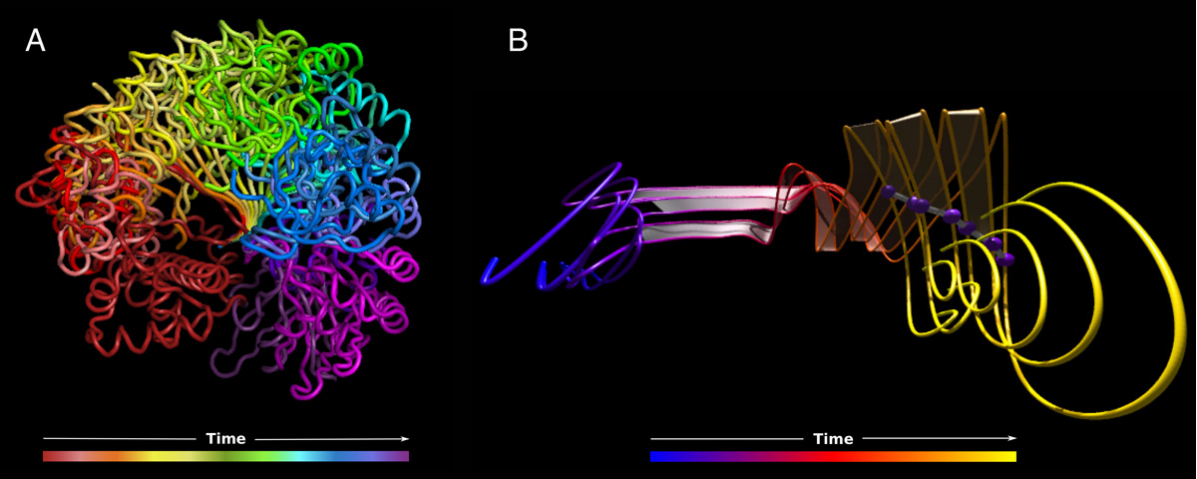
**MoFlow visualization reveals complex motions hidden by traditional molecular imagery**. Lid domain portion of a putative metal-dependent transcriptional regulator from *S. mutans*. (a) Cartoon-rendered (PyMOL) composite overlay view of the full protein at each time point in the simulation: red (beginning of trajectory) to purple (end of trajectory). (b) MoFlow rendering of six alpha carbons from the middle of the protein structure. These visualizations illustrate the contrast between the seemingly simple swinging motion in the composite image and the clearly more complex motion revealed by the pathline rendering.

Figure 3 shows two renderings of a predicted motion undergone by the lid domain of a putative metal-dependent transcriptional regulator from *Streptococcus mutans*. Figure 3a shows a canonical, non-MoFlow rendering of each step in the hinge-like motion of the protein domain, from brick-red on the left moving clockwise to purple on the right. In this composite, the motion seems quite simple, with the motile arm describing a smooth sweeping motion from left to right. The MoFlow rendering (Figure 3b) of a subset of atoms participating in this motion reveals that this simple interpretation is deceptive; the timeline representation provided in Figure 3a obscures a motion that is considerably more complex than a simple clockwise sweep.

**Figure 4:**
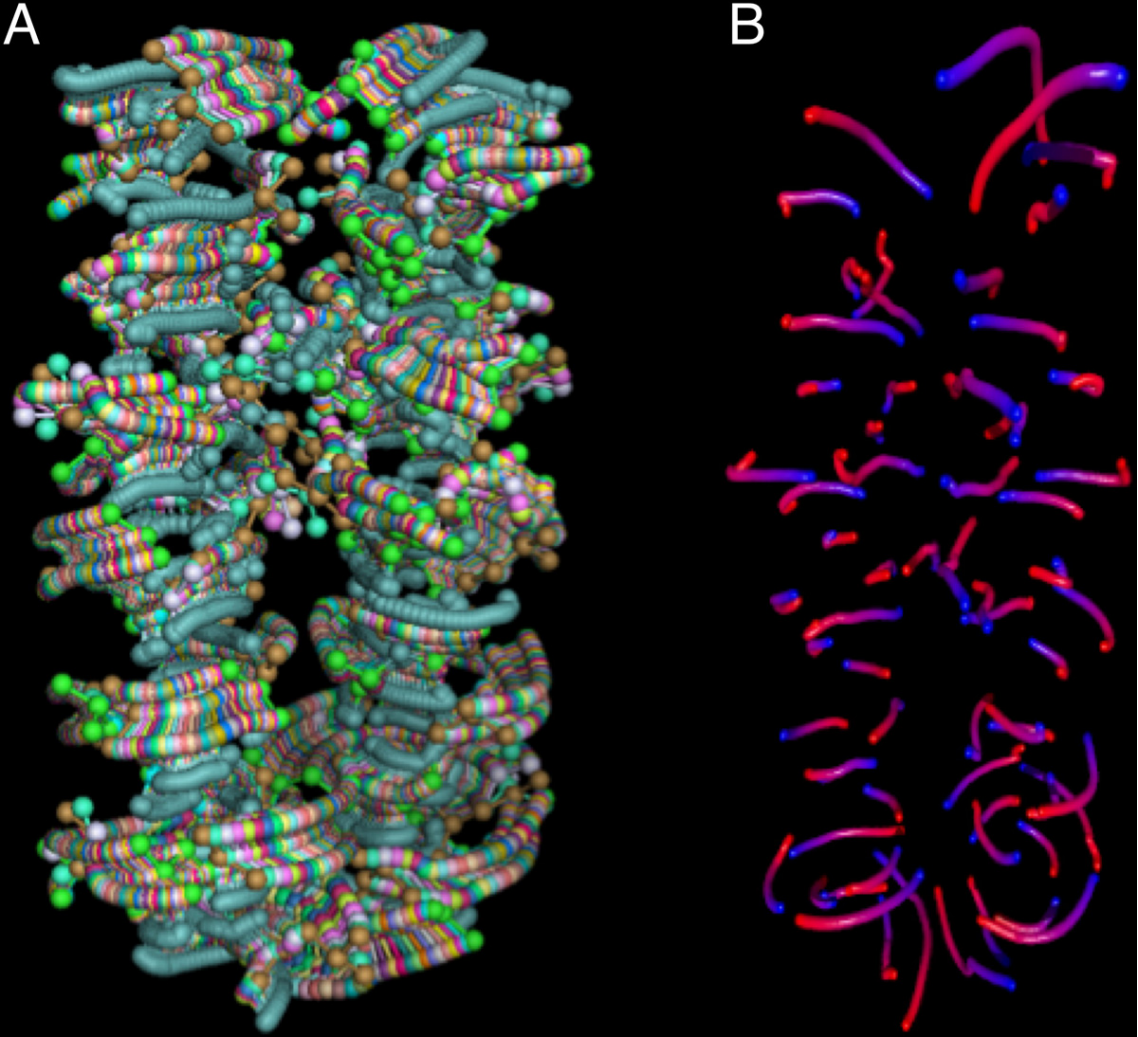
**Two non-connected α helices completing a half-turn rotation around their respective vertical axes**. (a) The simplicity of the motion is hidden by the composite overlay of the structures at each time-point. (b) Only the backbone alpha carbons along the helices as well as the gamma carbons from each of the Lysine residues. The short curves and blue-to-red (beginning/end) gradient clearly and efficiently describe the simple half-turn rotation undergone by both helices.

Figure 4 shows a different application of MoFlow. This figure displays two alpha helices that undergo rotation around their axes. Here, the backbones do not undergo significant conformational change, but the concerted motion of the sidechains is of interest. The canonical representation on the left (Figure 4a) presents the eye with a cacophonous display of atoms that do not intuitively present the eye with any suggestion of motion. The MoFlow representation on the right (Figure 4b) was created using the alpha and gamma carbons from the lysine residues that protrude from the helices. The resulting short blue-to-red gradient curves guide the viewer's eye to follow the rotational motion.

**Figure 5:**
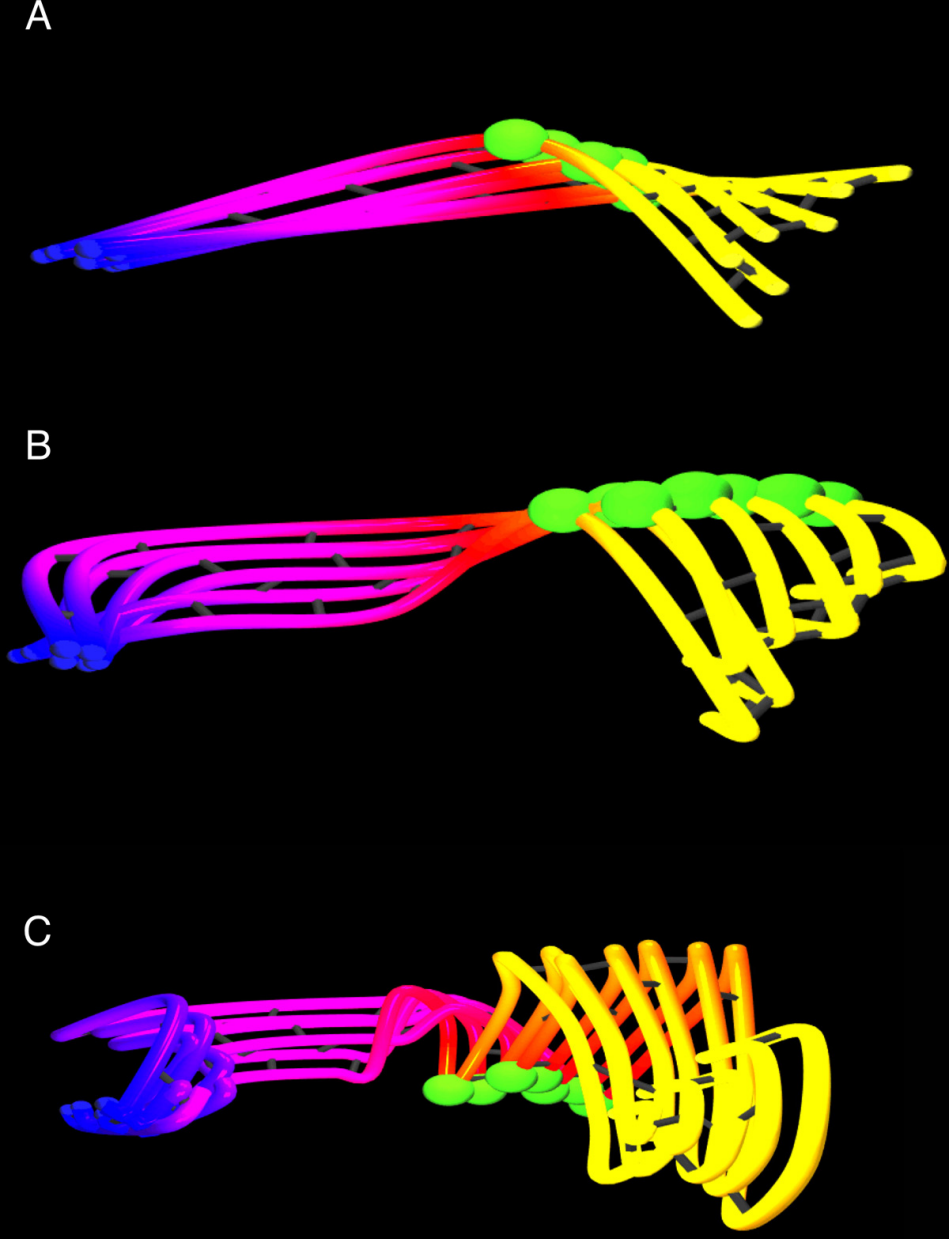
**The starting and ending poses are the same in each subfigure**. As more poses are used to construct the interpolating splines, more detail regarding the motion through the pose space is shown. While not shown here, if too many poses are used, the communication of motion again becomes problematic, as the representation becomes overwhelmed by random high-frequency perturbations from the molecular dynamics simulation. Automatic optimization of the number of poses displayed is a work in progress.

To aid in designing the final visual representation of motion, MoFlow allows the user to choose the number of "frames", or poses, of motion to include in the POVRay output. Figure 5 shows the MoFlow user interface. After uploading an appropriate multi-pose PDB file, the user can choose how many poses to include. The WebGL display updates to show a low-resolution preview of the rendering, allowing the user to experiment with the values before deciding on a final setting.

### Validation of approach

To validate the utility of MoFlow outside our original design intent of aiding in engineering AAV2, we have conducted an initial user survey comparing the ability of users with minimal expertise in molecular visualization to correctly identify key features of molecular motion when presented with either canonical, or MoFlow representations for the motion The preliminary analysis was aimed at addressing the first user task described above, e.g. "it moved like this". This preliminary data will be used to help design a larger study and are included here as an exploratory example; further validation studies are planned. The survey forms are available upon request from the authors.

The subject pool consisted of 12 subjects (4 female) aged between 13 and 48, from a variety of educational backgrounds and professions. All subjects had normal color vision. None had significant experience with molecular visualization.

Images were generated either as a composite (non-MoFlow) multi-pose rendering or as a MoFlow time lapse rendering. Poses in the canonical rendering were manually colored to match, as near as possible, the temporal coloring applied to the MoFlow timelines (time-gradient coloring is not a normal feature of existing molecular-visualization software).

Each subject was shown, in random order, the individual images depicting conformational change in molecules, without being led or informed as to the differences between them (e.g. composite poses vs MoFlow). Users were told that the images represented a conformational change where the molecule moved from one conformation to another, the temporal coloring scheme was explained, and the subjects were asked to briefly study each and then complete a series of questions for each image. These questions were both task-oriented - designed to identify the level of understanding conveyed by the image (e.g., could the user correctly map the starting, midpoint, and ending positions of specific components of the molecule) - as well as subjective (e.g., ease of use and time to completion). For each of three atoms in indicated at the midpoint of the molecular motion, a set of possible starting and ending points were labeled (both true and false targets) and users asked to select the beginning and ending target for each indicated atom.

To assess the utility of MoFlow we evaluated each users' position-mapping in a pair-wise fashion, where a correct mapping between the midpoint and any endpoint received a +1 score. The maximum score for each image was 6, as there were three atoms for which the users had to identify start and end points.

## Results

While this preliminary survey was only intended to guide our development of a more meaningful survey, rather than to reach statistical significance, overwhelming feedback from users indicated that the MoFlow visualizations could be understood more easily, more completely, and more quickly. Users were more likely to score higher (e.g., correctly identify the start and end points of motion) on the MoFlow diagram than on the conventional overlaid poses diagram. Individual user scores ranged from between 0 and 4 (out of a possible 6) for both visualization schemes, with an average score of 1.5 for the traditional representation and an average score of 2.67 for MoFlow. Several of the users presented with the canonical representation after having seen the MoFlow representation reacted with considerable annoyance at having to try to interpret the "mess". Users presented with the MoFlow representation after the canonical representation instead expressed relief that the "rollercoaster" (pathlines) helped them understand what they had been trying to interpret in the previous image; one user wrote that the MoFlow diagram "...was super easy compared to the alternative". Indeed, many users expressed that the canonical representation failed to present any indication of motion, whereas the MoFlow pathlines were more intuitively representative of motion. Qualitatively, the preliminary survey suggested that MoFlow was generally easier to use, with an average ease-of-use score of 4.58/10 (where higher is better), as opposed to 2.83/10 for the canonical representation.

## Discussion

We have presented a new method for rendering and visually exploring molecular motion that specifically takes into account the natural human intuition of movement over time. By indicating the motion of atoms as pathlines rather than the traditional orthogonal timeline representation, the eye is drawn naturally along the path of motion, providing an immediate understanding of the overall motion.

As demonstrated in Figures 2, 3, 4, 5, the MoFlow technique permits an intuitive understanding of molecular motion at any scale. This is supported by our preliminary user survey data, suggesting that on average users are likely to correctly identify molecular paths twice as accurately when presented with a MoFlow diagram.

**Figure 6:**
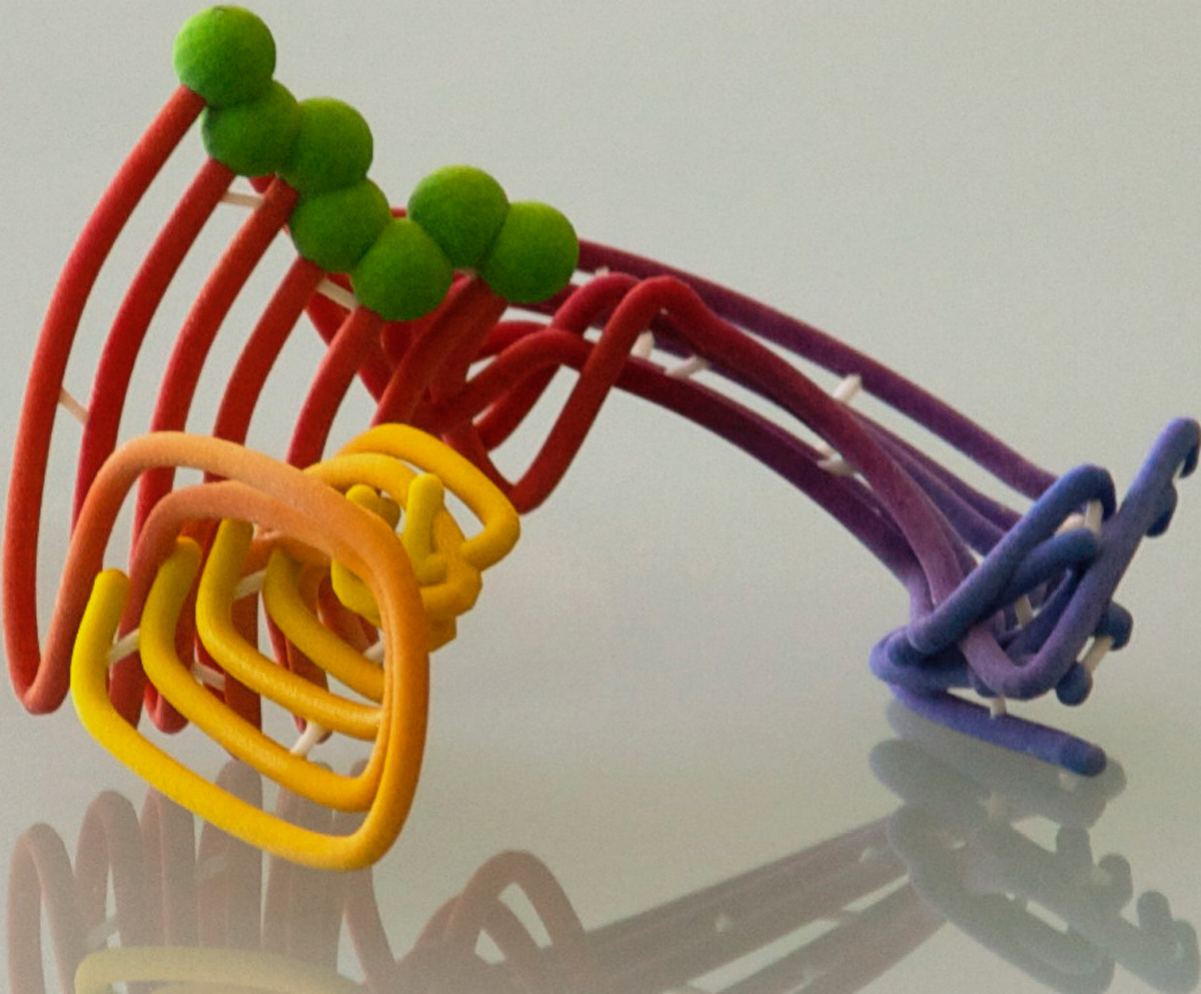
**3D color print (ZCorp Z650) of a MoFlow molecular motion rendering**.

MoFlow renderings are conveyed to the user as interactive WebGL objects as well as returned to the user by email in POV-Ray [15] format. The POV-ray version is suitable for high-resolution publication figures, and can be adapted to the user's needs by editing the camera position and other parameters. The WebGL version also enables export of a color-per-vertex VRML file that is compatible with most common 3D printing software. Figure 6 shows an example color 3D printout of the molecular motion shown in Figure 4B.

As with many other applications, the ability to engage the user's senses with a tactile model greatly appears to increase understanding of the process involved - in this case, allowing the user to freely rotate the object and observe it from any perspective provides greater insight than a 2D - or even on-screen interactive 3D - visualization. Comparison of the benefit of onscreen versus 3D-printed MoFlow visualization is an ongoing project.

In its current form, MoFlow satisfies the needs of the non-molecular-biologist end-user to qualitatively understand the trajectory features of molecular-motion data. The tasks as originally presented can be accomplished, and the visualizations were critical for the success of our AAV2 engineering project and aiding our gene-therapist collaborator to understand where to modify his molecule [13]. MoFlow has also proven indispensible to a collaborating virology lab in understanding the molecular rearrangement of the Respiratory Syncytial Virus Fusion protein during triggering (NIH R01 AI095684).

The MoFlow application is freely accessible via the web at http://www.mathmed.org/moflow/ and requires a multi-pose PDB file as input. Output is provided as a WebGL response that can be saved from the browser, a POV-Ray file that requires rendering prior to visualization, and optionally as a 3D-printable color-per-vertex VRML file. The authors welcome any comments and feedback on this application.

### Limitations and future work

The MoFlow can currently import and generate visualizations for large molecules undergoing complex conformational changes, however, there remain a number of improvements that are necessary to maximize convenience, and additional research is required to optimize some aspects of the visualization. For example, the user must currently manually balance model resolution with hardware limitations on the workstation - too many input poses, or requesting too-fine a tessellation in the WebGL version, can easily exhaust many system's available memory. In addition, it is not currently clear what the optimal temporal resolution for representing the motion in an MD simulation should be. Too fine a temporal resolution results in visualizations that are dominated by random perturbations of the structure and that fail to convey the overall conformation-change motion, while too coarse temporal resolution results in important aspects of the conformational change being omitted.

Because the motion paths are interpolated between the atom positions in the input poses, the implied bond-lengths at interpolated positions are likely to be inaccurate, and in some cases implausible. For MD simulations where successive poses are separated by 1 to 2 bond-lengths, in practice this interpolation error does not appear to produce significantly inaccurate paths. In the MoFlow interface, the molecular backbone chain can be constrained to any of the input poses to maintain accuracy. Alternatively it can be placed at interpolated positions between the predicted poses, with the understanding that this interpolated pose is also subject to interpolation errors.

**Figure 7:**
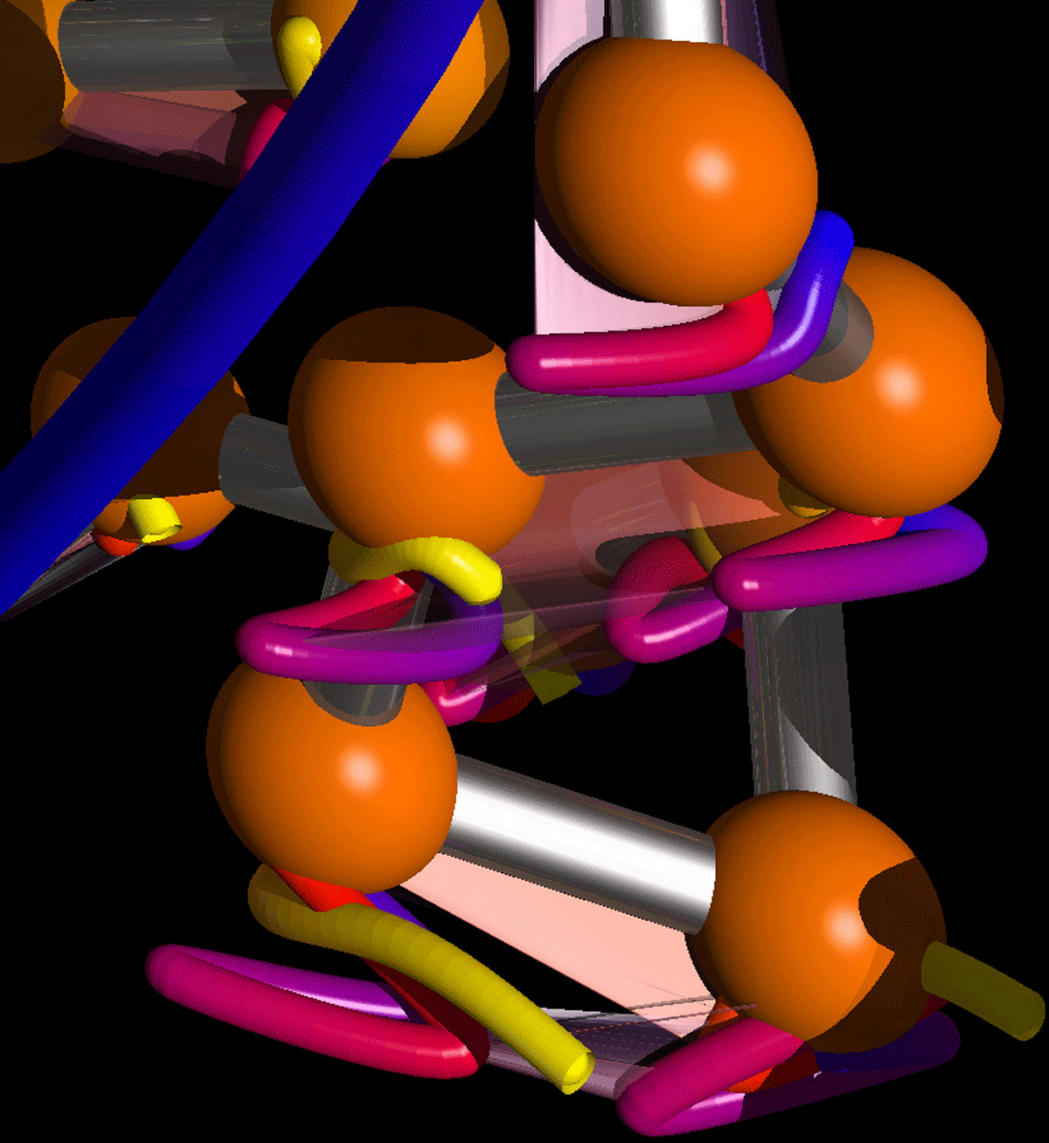
**Motion paths for thermal noise tend to wander randomly around each atom's location**.

The influence of thermal-motion noise in a simulation, coupled with the spline interpolation between input poses, can, in some circumstances, produce completely unexpected results. Typical brownian-motion effects appear as motion paths that "wander around" the atom positions, usually within 1 to 2 atomic radii, such as the paths shown in Figure 7. Due to the way that spline interpolation works, in rare cases a combination of input poses will cause small segments of the spline interpolation to make dramatic excursions away from the pose coordinates. The motion path appears to "blow up" into flower-petal-shaped loops. These are an inevitable consequence of using a continuously-smooth interpolation between positions, rather than linearly interpolating between poses, so they cannot be eliminated completely without losing smooth interpolation. However, we have only observed them a handful of times in several years of using MoFlow. They are easily eliminated by choosing slightly different input poses.

At the same time, it is clear that viewpoint and subselection of regions of the motion-structure to visualize, can maximize, or significantly impede the utility of the MoFlow representation (the same, of course, is true for any other representation scheme as well). We do not currently have an implemented solution for guiding the user to the most useful viewpoints and atom selections, however, preliminary attempts at maximizing the visually subtended surface area of the swept inter-pathline "ribbon" surfaces shows promise for selecting maximally informative views.

All of these issues together highlight the fact that the use and interpretation of MoFlow representations requires a careful user. We have found no algorithmic solution that can eliminate all of the possible visualization flaws automatically, without also eliminating details that some users want to see: We cannot arbitrarily low-pass filter the data to eliminate thermal noise, without impeding users who want to see where thermal noise is greatest, and where it is least in their system; we can't eliminate regions of the motion to de-clutter the display, without confusing users interested in relative motion (or lack thereof) in those regions, etc.

As a result, MoFlow requires the user to provide input data at a temporal and spatial resolution that they consider appropriate, and through the MoFlow interface we provide tools to enable the user to simplify or focus the visualization on the features they consider relevant. Currently the MoFlow interface enables the user to limit the range of poses used to a subset of all uploaded poses, limit the range of atoms of the molecule that are shown, reduce the density of alpha-carbons shown such that a motion path is shown for every n^th ^atom, adjust the position of the backbone along the motion paths, and adjust the viewpoint of the visualization. Many additional parameters can be adjusted by modifying variables within the renderable and 3D printable files that MoFlow returns. In addition, we encourage users to contact us with suggestions, as MoFlow remains a continually-improving platform.

Moving forward, in addition to improving the visualization and interface, and increasing automation for the user, we are pursing new avenues for comparative studies of molecular motion that are made possible by MoFlow representations. Because MoFlow represents *motion *as a structure, it becomes possible to compare the structures generated for different motions. This enables visualization of properties such as the uncertainly and randomness present in molecular motions in a way that is impossible for representations that rely on *molecular *structures to infer motion.

## Conclusions

We have described here a new method for rendering and visual exploration of molecular motion that specifically capitalizes on the natural human intuition of movement over time. The representation has been successfully used for its intended purpose to facilitate protein engineering. It has been preliminarily validated to convey more accurate information, more easily, to untrained consumers of molecular-motion data. Simultaneously the fact that this system directly represents motion as a structure, rather than inferring motion, creates opportunities for new types of molecular comparisons. The core of this system is representing the motion of atoms as pathlines, as opposed to the orthogonal timeline representation of traditional molecular motion imagery and animation. This correction of the representation to align with the actual data of interest provides advantages in both perception and in the variety of questions that can be asked of the data.

## Competing interests

The authors declare that they have no competing interests.

## Declarations

This work and its publication was funded in part by funds from the Research Institute at Nationwide Children's Hospital and in part by NSF project DBI-1262457 to WCR. SMD is supported by NIH/NIDCR grant T32DE014320.

RWR and SD contributed equally to the preparation of this manuscript as first authors.

AS contributed to the figures and the MoFlow code.

WCR conceptualized and implemented MoFlow, and directed and edited the manuscript.

This article has been published as part of *BMC Proceedings *Volume 9 Supplement 6, 2015: Proceedings of the 5th Symposium on Biological Data Visualization: Part 2. The full contents of the supplement are available online at http://www.biomedcentral.com/bmcproc/supplements/9/S6.
